# Lipoxin A_4_ and 15-Epi-Lipoxin A_4_ Protect against Experimental Cerebral Malaria by Inhibiting IL-12/IFN-γ in the Brain

**DOI:** 10.1371/journal.pone.0061882

**Published:** 2013-04-16

**Authors:** Nathaniel Shryock, Cortez McBerry, Rosa Maria Salazar Gonzalez, Steven Janes, Fabio T. M. Costa, Julio Aliberti

**Affiliations:** 1 ivisions of Cellular and Molecular Immunology and Pulmonary Medicine, Cincinnati Children's Hospital Medical Center, University of Cincinnati, College of Medicine, Cincinnati, Ohio, United States of America; 2 Department of Genetics, Evolution and Bioagents, Institute of Biology, University of Campinas, Campinas, Sao Paulo, Brazil; Université Pierre et Marie Curie, France

## Abstract

Cerebral malaria is caused by infection with *Plasmodium falciparum* and can lead to severe neurological manifestations and predominantly affects sub-Saharan African children. The pathogenesis of this disease involves unbalanced over-production of pro-inflammatory cytokines. It is clear that signaling though IL-12 receptor is a critical step for development of cerebral malaria, IL-12 genetic deficiency failed to show the same effect, suggesting that there is redundancy among the soluble mediators which leads to immunopathology and death. Consequently, counter-regulatory mediators might protect the host during cerebral malaria. We have previously showed that endogenously produced lipoxins, which are anti-inflammatory mediators generated by 5-lipoxygenase (5-LO)-dependent metabolism of arachidonic acid, limit host damage in a model of mouse toxoplasmosis. We postulated here that lipoxins might also play a counter-regulatory role during cerebral malaria. To test this hypothesis, we infected 5-LO-deficient hosts with *P. berghei* ANKA strain, which induces a mouse model of cerebral malaria (ECM). Our results show accelerated mortality concomitant with exuberant IL-12 and IFN-**γ** production in the absence of 5-lipoxygenase. Moreover, in vivo administration of lipoxin to 5-LO-deficient hosts prevented early mortality and reduced the accumulation of CD8^+^IFN-**γ** ^+^ cells in the brain. Surprisingly, WT animals treated with lipoxin either at the time of infection or 3 days post-inoculum also showed prolonged survival and diminished brain inflammation, indicating that although protective, endogenous lipoxin production is not sufficient to optimally protect the host from brain damage in cerebral malaria. These observations establish 5-LO/LXA_4_ as a host protective pathway and suggest a new therapeutic approach against human cerebral malaria (HCM). (255 words).

## Introduction

Cerebral malaria is a severe neurological complication of infection with *Plasmodium falciparum*. This disease predominantly occurs in children in sub-Saharan Africa, where approximately 500,000 people are affected annually, with fatality rates ranging from 17.5 to 19.2%. Moreover, approximately 25% of cerebral malaria survivors develop long-term neurological sequelae even after the appropriate antimalarial treatment [1]–[5].

The pathogenesis of cerebral malaria involves the sequestration of parasitized red blood cells in the brain microvasculature, the accumulation of mononuclear cells in brain tissue, and the increased expression of pro-inflammatory cytokines, including IFN-**γ** [6]–[22]. The large proportion of deaths that occurs in hospitals before anti-parasitic treatment can take effect highlights the importance of understanding the pathogenesis of this disease and of implementing new, rapidly acting interventions in combination with anti-plasmodium treatment [23]–[31].

Experimental cerebral malaria (ECM) caused by infection of C57BL/6 mice with *Plasmodium berghei* ANKA has been useful in identifying host factors involved in the pathogenesis of cerebral malaria and displays many features of the human disease[32]–[35]. Development of the mouse model requires an immune response against the parasites. Dendritic cells, CD4+ and CD8+ T cells, NK T cells, NK cells, and platelets have all been involved in disease induction and regulation. Additionally, while on one hand, IL-12 receptor is a critical component for the development of cerebral malaria, the use of single knockout mutants for several pro-inflammatory cytokines, including IL-12 have failed to show an obvious influence on cerebral malaria pathogenesis [6], [7], [9], [11], [14], [16], [18], [20], [22], suggesting that redundancy among those mediators might take place in vivo.

A balance between host pro-inflammatory and anti-inflammatory immune responses is a key determinant for the pathogenesis of cerebral malaria. Weaker pro-inflammatory responses could allow parasite persistence and proliferation, whilst exuberant pro-inflammatory responses could trigger lethal immunopathology, including cerebral malaria. Consequently, the identification of potent counter-regulatory pathways and mediators that control and/or inhibit the pathogenesis of cerebral malaria without promoting parasite proliferation and survival is important for the development of novel therapeutic interventions against this disease.

Lipoxins are a class of anti-inflammatory/pro-resolution lipid mediators derived from lipoxygenase-mediated metabolism of arachidonic acid. In recent years, a growing list of counter-regulatory actions has been attributed to lipoxins, including inhibition of chemotaxis, pro-inflammatory cytokine and chemokine production and NK cell activation, among others[36], [37].

5-lipoxygenase (5-LO), one of the enzymes required to generate lipoxin A_4_ (LXA_4_), is also needed to synthesize other mediators, such as leukotriene B_4_ (LTB_4_). Previously, we have used 5-lipoxygenase-deficient (*Alox5^−/−^*) mice to study the potential role of endogenously produced lipoxins in regulating the intensity and extent of pro-inflammatory response to infectious diseases, including toxoplasmosis and tuberculosis [38]–[40]. In hosts infected with these pathogens, we unveiled a lipoxin-triggered common regulatory mechanism–modulation of dendritic cell-IL-12 production. However, the outcome on immunopathology and survival after those infections depended on the nature of the pathogen. While the increased inflammatory response led to mortality in *T. gondii* infected mice, it triggered enhanced resistance to *M. tuberculosis*. Consistent with this, polymorphisms within the 5-lipoxygenase gene in humans are associated with resistance to tuberculosis in endemic areas in Africa, making it the first identified TB susceptibility gene in humans[41]. Lipoxins and its epimers (including 15-epi-LXA_4_) have also been shown to be produced in a 5-LO-independent manner in vivo after aspirin (via acetylated COX2) or statins (via S-nitrosylation of COX2)[42], [43]. 15-epi-LXA_4_ presented longer half-life in vivo when compared to LXA_4_ [44], nevertheless both molecules have shown overlapping biological actions.

Taking into account the intensity of inflammation during cerebral malaria and the results we observed during both *T. gondii* and *M. tuberculosis* infections, we hypothesized that the anti-inflammatory actions of lipoxins play a host-protective role during the pathogenesis of ECM. To test this hypothesis, we infected *Alox5* ^−/−^ mice with *P. berghei* ANKA strain. The results shown here indicate that endogenously generated LXA_4_ protects mice against ECM by inhibiting IL-12 production and accumulation of IFN-**γ**-producing cells in the brains of infected mice. In addition, we found that administration of 15-epi-LXA_4_ (a more stable endogenous epimer of LXA_4_) prolongs survival and dampens pro-inflammatory responses in *P. berghei*-infected WT mice. These observations provide a proof-of-concept for a potential new therapy for cerebral malaria in humans (HCM).

## Results

### 5-LO-deficient mice present accelerated mortality after *P. berghei* ANKA infection

**Figure 1:**
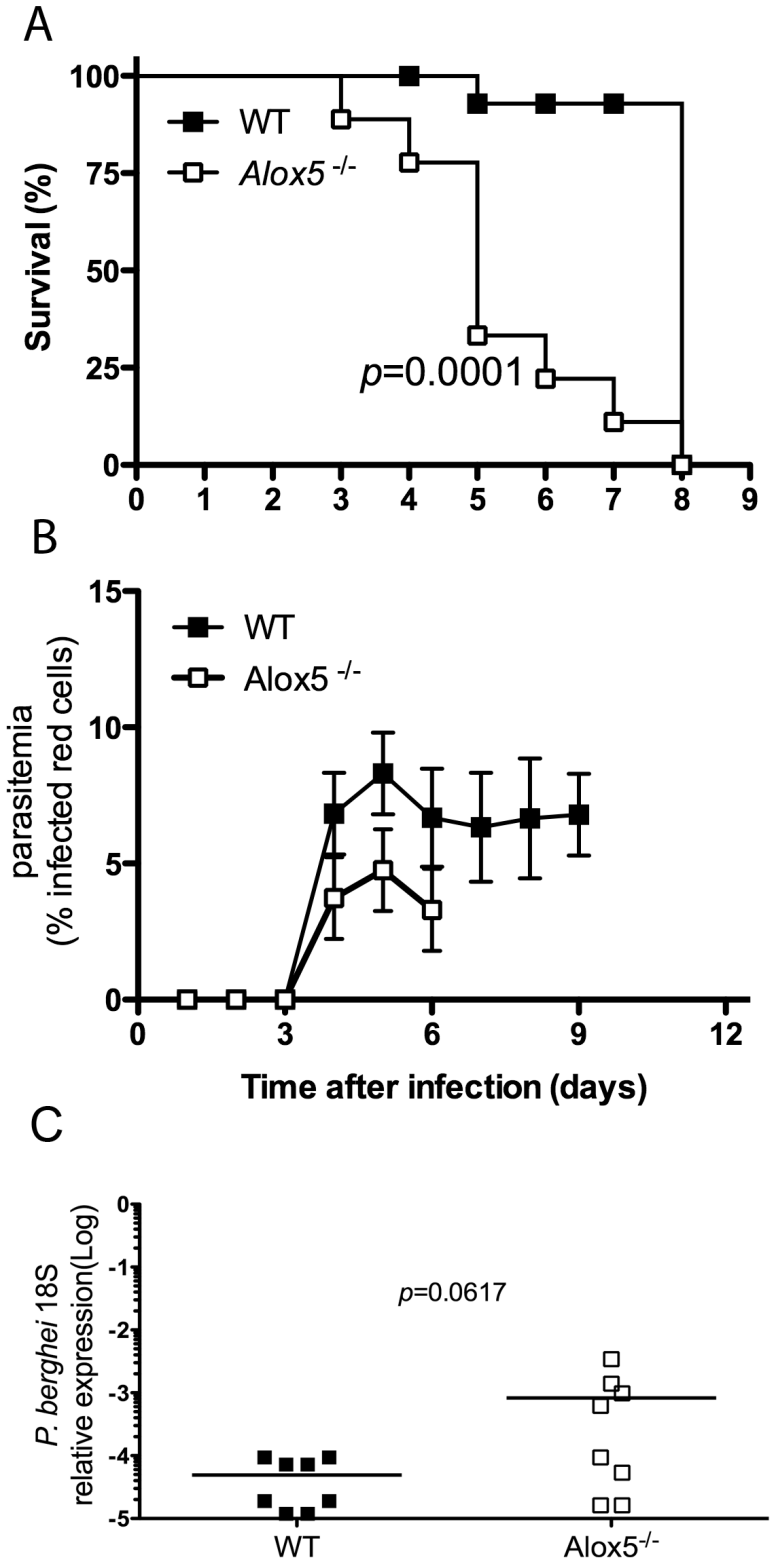
*P. berghei* ANKA infection causes accelerated mortality in 5-LO-deficient mice. C57Bl/6 WT or *Alox5* ^−/−^ (*n* = 8 mice/group) mice were infected i.p. with *P. berghei* ANKA strain. Survival (**A**) and parasitemia (**B**) were monitored daily. The presence of parasite 18S rRNA in the brains of infected mice was determined by real-time RT-PCR in perfused samples obtained 5 days after infection (**C**). Data are representative of 5 independent experiments performed with similar results. Statistical analysis of survival studies was determined using Log-rank (Mantel-Cox) method, in panel **C**, statistical analysis was performed using Mann Whitney test.

Cerebral malaria induced by *P. berghei* ANKA infection is typically characterized by intense CNS cellular infiltration with vascular and tissue damage, despite relatively low levels of parasitemia. Given the intensity of the inflammatory response, we hypothesized that 5-lipoxygenase-dependent arachidonic acid metabolism might either contribute to the severity of the disease, via synthesis of leukotrienes, or mediate host protective responses, via production of lipoxins. To distinguish between these possibilities, we infected both WT and *Alox5* ^−/−^ mice with *P. berghei* ANKA-parasitized red cells. Mean survival time (MST) was 8 days for WT mice, but only 3 days for *Alox5^−/−^* mice (**Figure 1A**). In contrast, parasitemia levels were similar in infected WT and *Alox5* ^−/−^ mice (**Figure 1B**). On the other hand, we found a trend for reduction in the levels of parasite 18S rRNA in the CNS at 5 days after infection (**Figure 1C**), therefore excluding the possibility that the more severe pathology is associated with increased parasite sequestration. Thus, 5-lipoxygenase may contribute to host survival by limiting inflammation rather than by limiting parasite proliferation or survival.

### 5-LO-dependent control of IL-12p70 and IFN-γ during *P. berghei* ANKA infection

**Figure 2:**
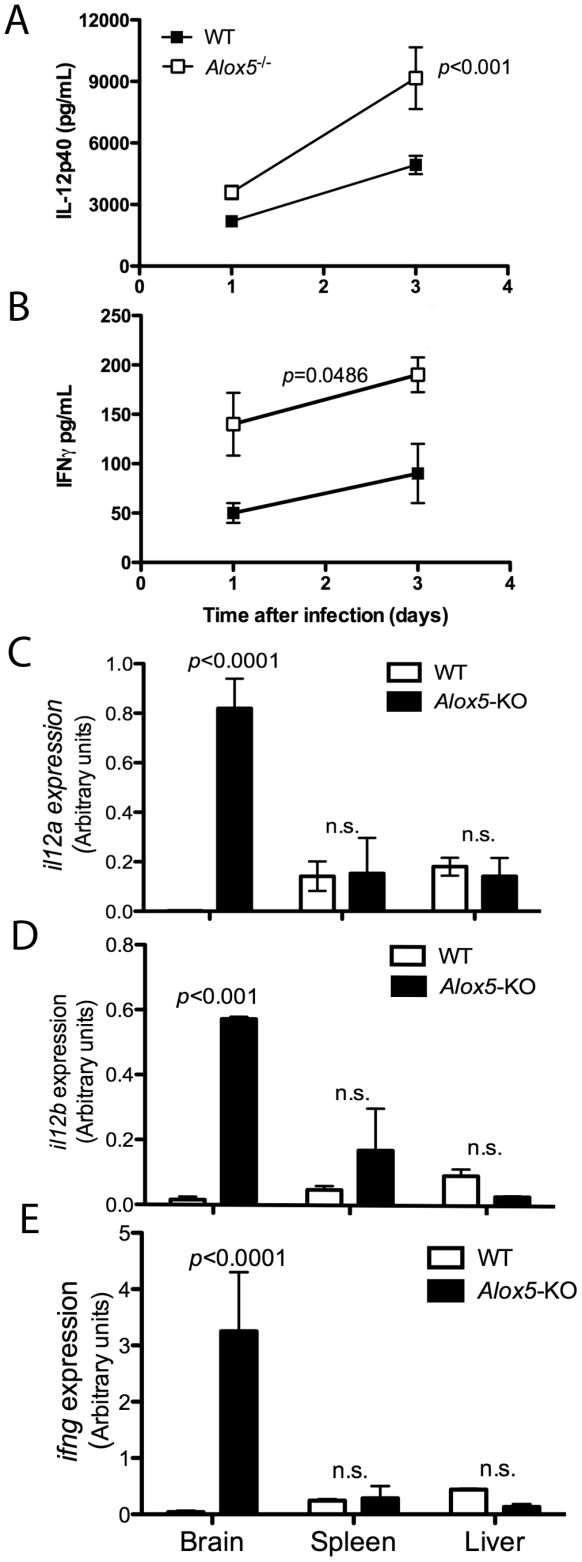
Enhanced cytokine expression during *P. berghei* ANKA infection in 5-LO-deficient mice. C57Bl/6 WT and *Alox5* ^−/−^ (*n* = 4 mice/group) mice were infected i.p. with *P. berghei* ANKA strain. At 1 and 3 days after infection, animals were bled and serum levels of IL-12p40 (**A**) and IFN-**γ** (**B**) were determined by ELISA. Five days after infection, mice were sacrificed and brains, livers and spleens harvested. Tissues were homogenized, total RNA extracted and reverse transcripted for real-time RT-PCR determination of *il12a* (**C**), *il12b* (**D**) and *ifng* (**E**) expression. Data shown are representative of one out of three independent experiments performed. Statistical differences were determined using Mann Whitney test.

Type 1 cytokines, including IL-12 and IFN-**γ**, are associated with murine cerebral malaria pathogenesis. Despite its protective role during the liver stage of infection, IFN-**γ** can damage the host during blood stage of severe forms of malaria, including cerebral malaria. We hypothesized that counter-regulatory pathways might be required to limit host damage caused by an overly exuberant type 1 cytokine response. Consequently, we tested whether the accelerated mortality of *Alox5* ^−/−^ mice might be due to higher levels of IL-12 and IFN-**γ** after *P. berghei* ANKA infection. *Alox5* ^−/−^ mice had significantly increased serum levels of both cytokines 3 days after infection, as compared to WT mice (**Figure 2**). Consistent with this, *il12a*, *il12b*, *ifng*, *il6*, *il17a* mRNA expression were considerably increased in the brains of *P. berghei* ANKA-infected Alox5-KO vs. WT mice at 5 days after infection (**Fig. 2C-E** and **figure S1**).

### 5-LO-mediated synthesis of LXA_4_ during ECM

**Figure 3:**
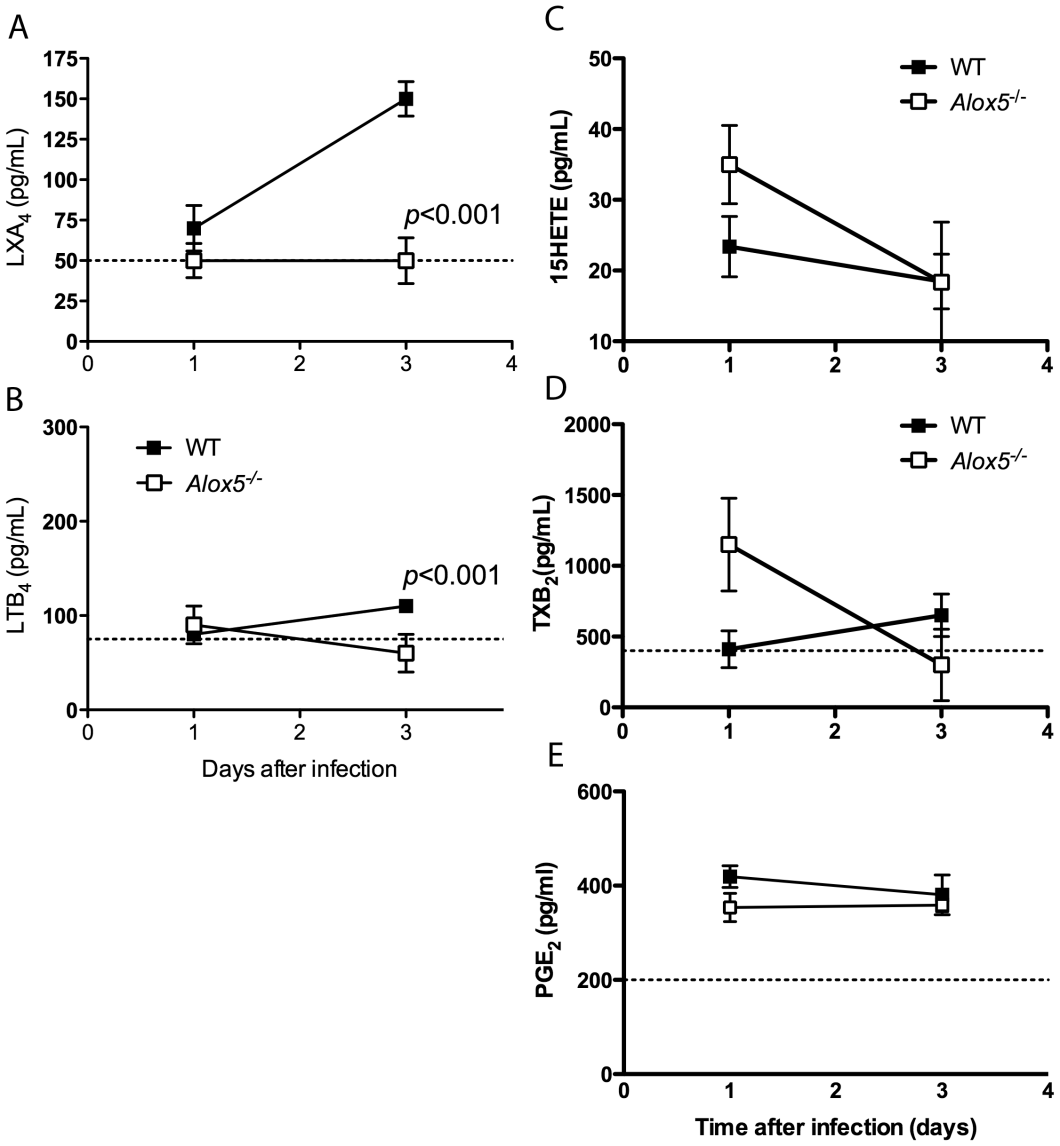
*P. berghei* ANKA infection induces LO-dependent lipid mediator synthesis. C57Bl/6 WT and *Alox5* ^−/−^ (*n* = 4 mice/group) mice were infected i.p. with *P. berghei* ANKA strain. At 1 and 3 days after infection, animals were bled and serum levels of LXA_4_ (**A**), LTB_4_ (**B**), 15HETE (**C**), TXB_2_ (**D**) and PGE_2_ (**E**) were determined by ELISA. Data shown is representative of one out of three independent experiments performed. Statistical analysis of difference among groups was determined using Mann Whitney test.

The results shown so far indicate that the absence of 5-lipoxygenase led to aberrant IL-12 and IFN-**γ** production during ECM, suggesting a potential defective counter-regulatory pathway. Because 5-lipoxygenase mediates the synthesis of several arachidonic acid-derived lipid mediators, including leukotrienes and lipoxins, we investigated whether genetic deficiency of 5-lipoxygenase would alter the profile of arachidonic acid-derived mediators. Serum LTB_4_ and LXA_4_ levels were significantly reduced, while PGE_2_ and 15-HETE levels were not significantly altered by 5-lipoxygenase deficiency during *P. berghei* ANKA infection (**Figure 3A–E**). Interestingly, serum TXB_2_ concentration was increased 3, but not 5 days after infection. Taking together, the data indicate that 5-lipoxygenase mediates synthesis of LXA_4_ and LTB_4_ during *P. berghei* ANKA infection without significantly affecting the levels of other lipid mediators.

### Increased CD4+ and CD8+T cell infiltration, IL-12+ and IFN-γ+ cells in infected 5-LO-deficient hosts

**Figure 4:**
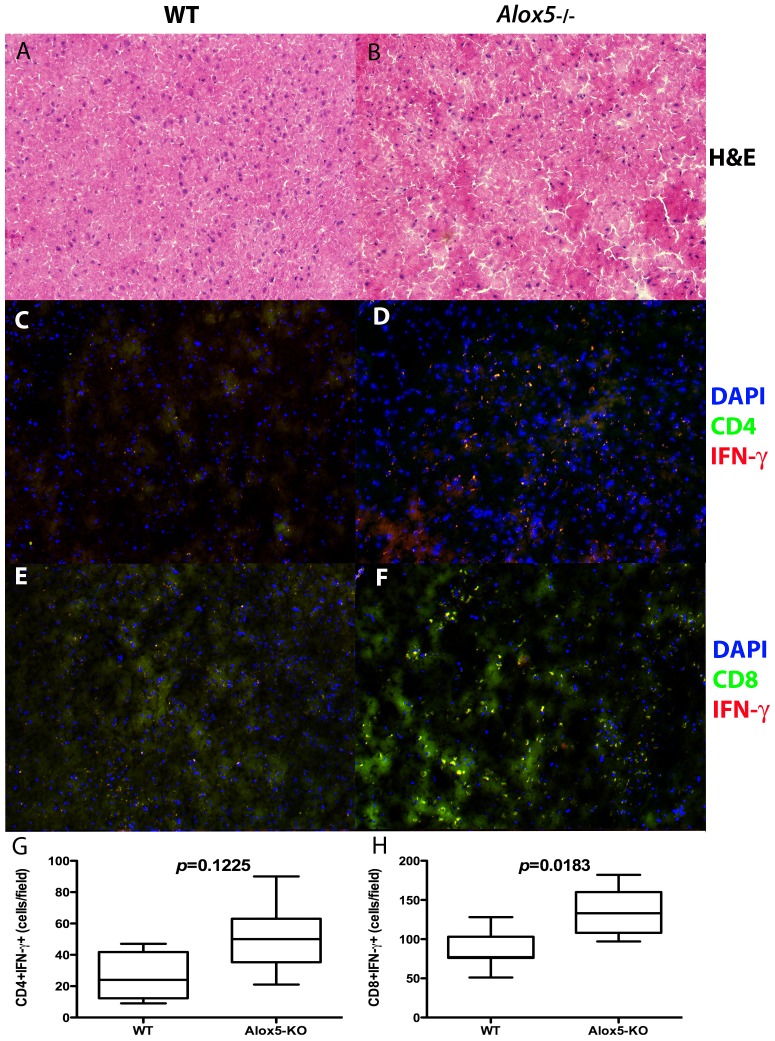
Increased inflammation and CD8+ IFN-γ + cells in brains from *P. berghei* ANKA-infected *Alox5* ^−/−^ mice. C57Bl/6 WT (A, C and E) and *Alox5* ^−/−^ (B, D and F) (*n* = 4 mice/group) mice were infected i.p. with *P. berghei* ANKA strain. At 5 days after infection, animals were sacrificed, brains harvested and tissue sections obtained. Panels **A** and **B** show H&E micrographs. **C** and **D** show immunofluorescence staining for CD4 (green) and IFN-**γ** (red). **E** and **F** show staining for CD8 (green) and IFN-**γ** (red). DAPI was used as a counterstain (**C–F**). Double-positive (CD4+ IFN-**γ**+−**G**, and CD8+ IFN-**γ**+−**H**) cells were quantified using ImageJ software. Magnification 200x. Statistical analysis in data from panels **E** and **F** was performed using Mann-Whitney test.

The increased mRNA and protein levels of both IFN-**γ** and IL-12 in *P. berghei* ANKA-infected *Alox5^−/−^* mice suggested that the lack of an endogenous 5-LO-dependent anti-inflammatory pathway led to either higher cytokine production by pathogen-specific cells or increased accumulation/proliferation of cytokine producing cells along the perivascular areas of the brains of infected mice. To evaluate this possibility, we enumerated CD4+ and CD8+T cells, as well as IFN-**γ**+ cells in the brains of *P. berghei* ANKA-infected WT and *Alox5^−/−^* mice 5 days after infection (**Figure 4A–B**). Increased frequency of densely stained areas surrounded by foamy weakly stained tissue was noted in brain sections from *P. berghei* ANKA-infected *Alox5^−/−^* mice, suggesting tissue damage. Although increased frequency of CD4+IFN-**γ**+ cells in brains of both WT and *Alox5^−/−^* infected mice did not differ significantly (**Fig. 4C, D and G** **)**, there was increased detection of CD8+IFN-**γ**+ cells in the brains of infected *Alox5^−/−^* versus WT mice (*p* = 0.0183) (**Fig. 4E, F and H**). Our additional observation that CD8+ IFN-**γ**-producing cells are more numerous than CD4+ IFN-**γ**-producing cells in the brains of infected mice (**Fig. 4G and H**) is consistent with previous reports of the presence of CD8+ T cell IFN-**γ** response in *P. berghei* ANKA-infected C57Bl/6 mice [8], [10], [12].

### 15-epi-Lipoxin A_4_ treatment prevents the onset of experimental cerebral malaria

**Figure 5:**
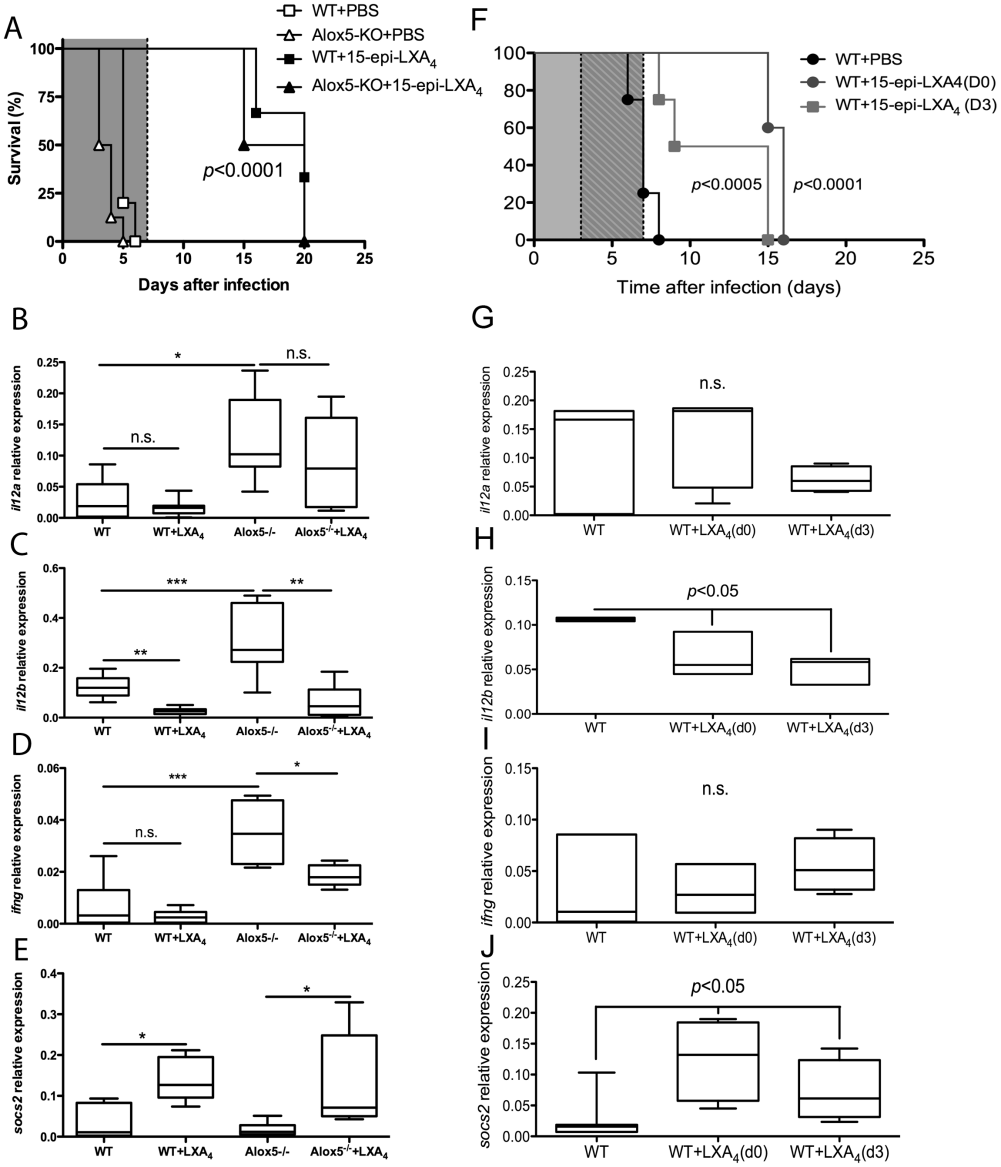
Exogenous delivery of 15-epi-LXA_4_ prolongs survival and reduces pro-inflammatory cytokine production of both WT and 5-LO-deficient mice after *P. berghei* ANKA infection. C57Bl/6 WT and *Alox5* ^−/−^ (*n* = 8 mice/group) mice were infected i.p. with *P. berghei* ANKA strain and treated with PBS alone or with 15-epi-LXA_4_ (1 µg/mouse) from day 1 to 7 after infection (shaded area in A and F) or from day 3 to 7 after infection (striped area in F). Survival was monitored (A and F) up to 20 days after inoculum. Five days after infection, mice were sacrificed and brains harvested. Tissues were homogenized, total RNA extracted and reverse transcribed for real-time RT-PCR determination of *il12a* (B and G), *il12b* (C and H), *ifng* (D and I) and *socs2* (E and J) expression. Data shown boxes/mean bar with min-max whiskers and are representative of three independent experiments performed. (*  =  *p*<0.05, **  =  *p*<0.01 and ***  =  *p*<0.0001) as determined by One-way ANOVA with Tukey multiple comparison test.

The results presented so far indicated that endogenous 5-LO provides some protection against *P. berghei* ANKA infection. Earlier studies have shown that lipoxins can promote resolution by inducing anti-microbial peptides and subsequent bacterial killing and clearance [45], [46]. To determine whether the effect seen in the absence of Alox5 is resulting from production of anti-inflammatory lipoxins, such as LXA_4_, we investigated whether in vivo delivery of 15-epi-LXA_4_ to mice could prolong survival while reducing type 1 cytokine production after *P. berghei* ANKA infection. Our results show that lipoxin treatment at the time of infection significantly prolonged survival of both infected *Alox5^−/−^* mice (MST 17.5 *vs*. 3.5 days) and WT mice (MST 20 vs. 5 days) for 15-epi-LXA_4_-treated and PBS-treated groups, respectively (**Figure 5A**). In agreement with our previous findings and with the changes in survival rates, 15-epi-LXA_4_ treatment lowered brain *il12a*, *il12b* and *ifng* mRNA expression in infected WT and *Alox5^−/−^* mice (**Figures 5B–D**), concomitant with increased expression of *socs2* mRNA (**Figure 5E**). Thus, despite its production during *P. berghei* ANKA infection, increased lipoxin levels are beneficial to the host, diminishing the severity of the pro-inflammatory response and prolonging survival. In order to further support the therapeutic potential of lipoxin-based interventions during cerebral malaria, we compared whether delayed treatment (starting 3 days after inoculum) would affect survival and cytokine mRNA profile of PBS-treated infected WT mice. In fact, as can be seen in **figure 5F**, mice that received 15-epi-LXA_4_ from day 3 through 7 after infection, presented a significant delay of mortality rates (gray squares) when compared to PBS-treated WT controls (black circles). Notably, the mortality rates did not significantly differ whether treatment with 15-epi-LXA_4_ initiated at the day of infection or three days later. The levels of *il12a* and *ifng* mRNA expression in the brain were not significantly different among the experimental and control groups (**Figures 5G and 5I**). On the other hand, treatment with 15-epi-LXA_4_ either at the time of infection or three days post-inoculum caused a significant reduction in the expression of *il12b* (**Figure 5H**), while significantly increased the expression levels of *socs2* (**Figure 5J**). While CNS *ifng* expression was dramatically reduced in Alox5^−/−^ mice after 15-epi-LXA_4_ (**Figure 5D**), both forms of 15-epi-LXA_4_ treatment failed to significantly change its expression levels in WT mice (**Figure 5D and 5I**). Taken together this set of results support that treatment 15-epi-LXA_4_ diminishes expression of some pro-inflammatory mediators and prevents mortality due to cerebral malaria even when mediator delivery is initiated three days after infection, thus providing support for the potential development of a novel supportive therapeutic venue that may prolong patient survival for sufficient time to allow anti-malarial drugs to take effect.

## Discussion

Cerebral malaria is a lethal severe form of the disease whose pathogenesis is complex and incompletely understood. Observations from animal models (e.g.; *P. berghei* ANKA infection in mice) and *P. falciparum*-infected humans support a hypothesis that sequestration of parasitized red cells by the brain microvasculature leads to vascular obstruction, edema, leukocyte activation and extravasation, hypoxia and necrosis. Although the precise phenotype of cerebral malaria can vary considerably, it is widely agreed that the pro-inflammatory cytokines, such as IFN-**γ** contribute to its pathogenesis and clinical features [1], [3], [6], [7], [9], [10], [12], [19]–[22], [32]–[34].

Activation of brain endothelial cells, infected red cells and circulating leukocytes by both IFN-**γ** and TNF during cerebral malaria most likely triggers the cascade of events that swiftly transforms the brain microenvironment. Consequently, these pro-inflammatory cytokines may well determine the intensity of tissue damage and, subsequently, the severity of the disease. With this in mind, we postulated that parasite-triggered host IFN-**γ** production is modulated by anti-inflammatory/counter-regulatory pathways. Our results show that ECM induces production of LXA_4_, a 5-lipoxygenase-derived arachidonic acid metabolite that dampens IL-12/IFN-**γ** production during infection with any of several pathogens [47] and up-regulates production of proteins that interfere with inflammatory cytokine signaling, such as SOCS2 [48] and promotes resolution[49].

The amount of LXA_4_ produced during infection influences host survival. On one end of the spectrum, the absence of LXA_4_ production during infection with the highly virulent parasite, *Toxoplasma gondii*, increases production of IL-12 and IFN-**γ** and exacerbates inflammation, resulting in host death from encephalitis [39]. On the other end of the spectrum, enhancement of IL-12 and IFN-**γ** production by LXA_4_ deficiency protects hosts infected with *M. tuberculosis* by increasing clearance of the bacilli from the lungs [40]. Consistent with this, clinical studies in *M. tuberculosis* endemic areas associate *ALOX5* promoter polymorphisms that decrease gene expression with a lower frequency of active disease [41]. Thus, by regulating inflammation that, in different situations can protect the host by suppressing the pathogen or kill the host by damaging essential organs, LXA_4_ may determine infection outcome.

The results shown here establish that *alox5*-dependent LXA_4_ production modulates disease severity in *P. berghei*-infected mice. Absence of LXA_4_ production during *P. berghei* ANKA infection was associated with higher systemic levels of IL-12 and IFN-**γ** and increased *il12a, il12b* and *ifng* mRNA expression in the brain. Importantly, the accelerated mortality of *alox5*-deficient mice could be prevented by in vivo delivery of 15-epi-LXA_4_. This is a critical observation, because Alox5 is involved in the synthetic pathways of several arachidonic acid-derived mediators, including LTB_4_. Consistent with this, *alox5*-deficient mice failed to produce both LXA_4_ and LTB_4_ after infection, although serum levels of other arachidonic acid metabolites, including PGE_2_, TXB_2_ and 15HETE, were not affected. However, the residual production of LXA_4_ detected here suggests that alternative pathways play a minor, but detectable role in the generation of LXA_4_.

Furthermore, in support to a protective role of lipoxins, in vivo delivery of 15-epi-LXA_4_ to infected WT mice significantly prolonged survival beyond the ECM mortality period, while *reducing il12b* mRNA expression levels in the brain. The effects of 15-epi-LXA_4_ treatment did not significantly reduced *ifng* expression in WT mice, yet the treatment showed protection against ECM mortality. It is possible that lipoxins promoted protection via inhibition of cytokine-mediated host damage. This more targeted effect could explain the apparent discrepancy between the weak inhibition of *ifng* expression while significantly prolonging survival. The protective results with 15-epi-LXA_4_ treatment in WT mice indicates that the endogenous production of LXA_4_ is not sufficient to optimally protect the host from inflammatory brain damage during cerebral malaria and suggests that genetic variability in LXA_4_ production may influence the risk of developing cerebral disease or other severe forms of malaria. If so, selective pressures that may favor increased LXA_4_ production, such as during *T. gondii* and *P. falciparum* infection, and selective pressures that may favor decreased LXA_4_ production, such as *M. tuberculosis* infection, could interplay in order to maintain considerable genetic diversity in regulation of 5-LO production in areas where all of these pathogens are endemic. Another closely related mediator that could potentially be involved here is LXB_4_ that has been shown to be equally active in vivo when compared to LXA_4_ [50]. However, further studies are required to address whether this mediator is produced and biologically active during ECM.

Our observations also suggest the possibility of using LXA_4_ stable analogs [51] as part of a therapeutic approach for severe malaria. Although such agents would not cure infection, they may prolong host survival sufficiently for traditional anti-malarial chemotherapeutics to effect a cure. A similar strategy has been attempted with corticosteroids [52]–[57] and aspirin [58], [59], with little or no success. Some aspirin-dependent anti-inflammatory actions are mediated by aspirin-triggered lipoxins [60]. Although aspirin also initiates several lipoxin-independent pathways, including suppression of prostaglandin production and platelet function, our results presented here provide support that lipoxins can provide a more targeted effect in controlling inflammation during ECM and, potentially HCM, as compared to those treatments previously tested. Consequently the potential use of lipoxins and its analogs in a more targeted approach against HCM is likely to promote survival and is worthy of investigation.

## Materials and Methods

### Mice

C57Bl/6J and 5-lipoxygenase-deficient (*Alox5* ^−/−^) mice were bred and maintained in a specific pathogen free animal facility at Cincinnati Children's Hospital Medical Center. All procedures shown here were reviewed and approved by the Cincinnati Children's Hospital Medical Center Institutional Animal Care and Use Committee.

### Parasites and infections

*P. berghei* ANKA strain clone c115cy1 was maintained by continuous passage in vivo. For experimental infections, mice were inoculated with 10,000 infected red blood cells by intraperitoneal injection. Parasitemia was determined by counting infected red blood cells in Wright-Giemsa-stained blood smears.

### Cytokine and lipid mediators and ELISA kits

15-epi-LXA_4_ was obtained from Cayman Chemicals. Due to its labile nature, long-term storage stock solution was kept at −80°C. For in vivo treatments stock aliquots were diluted in PBS immediately at the time of use. IL-12p70, IL-12p40 and IFN-**γ** levels were measured using commercial ELISA kits (BD biosciences). LTB_4_, 15HETE, PGE_2_ and TXB_2_ ELISA kits were from Cayman Chemicals and an LXA_4_ ELISA kit was from Oxford. Detection limits for these assays were: 15 pg/mL (IL-12p40), 39 pg/mL (IL-12p70), 31 pg/mL (IFN-**γ**), 13 pg/mL (LTB_4_), 11 pg/mL (TXB_2_), 170 pg/mL (15HETE), 36 pg/mL (PGE_2_) and 20 pg/mL (LXA_4_) For *in vivo* experiments, infected mice (*n* = 4–8) were bled for assessment of plasma cytokine and lipid mediator levels.

### Real-time RT-PCR

Total RNA was isolated from tissues using the Trizol LS reagent according to the instructions of the manufacturer. cDNA was synthesized with TaqMan Reverse Transcriptase (Applied Biosystems, Foster City, CA) and mRNA expression of cytokines (IL-12p35, IL-12p40, IL-23p19, IFN-**γ**, IL-6, IL-17A and IL-23p19)–and β-actin were analyzed by RT-PCR. *P. berghei* 18S expression levels were determined in perfused brains harvested 5 days after infection. Real-time RT-PCR was performed on an ABI-Prism 7000 PCR cycler (Applied Biosystems).

### Microscopy

Brains were removed from mice up to 7 days after infection, and frozen sections were processed and stained with a anti-mouse IL-12p40, IFN-**γ**, CD4 and CD8 Abs, followed by a double incubation with Alexa Fluor 488- or 594-conjugated antibodies (Invitrogen). The slides were counterstained for nuclei with DAPI (Invitrogen). Images were acquired using a microscope (Axiovert, Carl Zeiss MicroImaging, Inc.) with the AxioVision software (Carl Zeiss MicroImaging, Inc.) and analyzed using ImageJ software.

### Statistical analysis

The statistical significance of differences in mean values between experimental versus control or vehicle treated samples was evaluated using the methods indicated in the figure legends. Differences were considered to be significant at *p*<0.05 unless otherwise indicated.

## Supporting Information

Figure S1 **Enhanced** ***il6*** **and** ***il17A*** **mRNA expression in** ***P. berghei*** **ANKA infected Alox5-deficient mice.** C57Bl/6 WT and *Alox5* ^−/−^ (*n* = 4 mice/group) mice were infected i.p. with *P. berghei* ANKA strain. Five days after infection, mice were sacrificed and brains, livers and spleens harvested, homogenized, total RNA extracted and reverse transcripted. Real-time RT-PCR was performed for determination of *il6* (**A**), *il23a* (**B**) and *il17a* (**C**) expression. Data shown are representative of one out of three independent experiments performed. Statistical differences were determined using Mann Whitney test.
(TIF)

## References

[1] Idro R, Jenkins NE, Newton CR (2005) Pathogenesis, clinical features, and neurological outcome of cerebral malaria. Lancet Neurol 4: 827–840. 16297841 10.1016/S1474-4422(05)70247-7

[2] Dondorp A, Nosten F, Stepniewska K, Day N, White N, et al (2005) Artesunate versus quinine for treatment of severe falciparum malaria: a randomised trial. Lancet 366: 717–725. 16125588 10.1016/S0140-6736(05)67176-0

[3] John CC, Bangirana P, Byarugaba J, Opoka RO, Idro R, et al (2008) Cerebral malaria in children is associated with long-term cognitive impairment. Pediatrics 122: e92–99. 18541616 10.1542/peds.2007-3709 PMC2607241

[4] John CC, Panoskaltsis-Mortari A, Opoka RO, Park GS, Orchard PJ, et al (2008) Cerebrospinal fluid cytokine levels and cognitive impairment in cerebral malaria. Am J Trop Med Hyg 78: 198–205. 18256412 PMC2254318

[5] Dondorp AM, Fanello CI, Hendriksen IC, Gomes E, Seni A, et al (2010) Artesunate versus quinine in the treatment of severe falciparum malaria in African children (AQUAMAT): an open-label, randomised trial. Lancet 376: 1647–1657. 21062666 10.1016/S0140-6736(10)61924-1 PMC3033534

[6] Villegas-Mendez A, Greig R, Shaw TN, de Souza JB, Gwyer Findlay E, et al (2012) IFN-gamma-producing CD4+ T cells promote experimental cerebral malaria by modulating CD8+ T cell accumulation within the brain. J Immunol 189: 968–979. 22723523 10.4049/jimmunol.1200688 PMC3393641

[7] Fauconnier M, Palomo J, Bourigault ML, Meme S, Szeremeta F, et al (2012) IL-12Rbeta2 is essential for the development of experimental cerebral malaria. J Immunol 188: 1905–1914. 22238458 10.4049/jimmunol.1101978

[8] Lau LS, Fernandez Ruiz D, Davey GM, de Koning-Ward TF, Papenfuss AT, et al (2011) Blood-stage Plasmodium berghei infection generates a potent, specific CD8+ T-cell response despite residence largely in cells lacking MHC I processing machinery. J Infect Dis 204: 1989–1996. 21998471 10.1093/infdis/jir656

[9] Villegas-Mendez A, de Souza JB, Murungi L, Hafalla JC, Shaw TN, et al (2011) Heterogeneous and tissue-specific regulation of effector T cell responses by IFN-gamma during Plasmodium berghei ANKA infection. J Immunol 187: 2885–2897. 21880980 10.4049/jimmunol.1100241 PMC3173971

[10] Claser C, Malleret B, Gun SY, Wong AY, Chang ZW, et al (2011) CD8+ T cells and IFN-gamma mediate the time-dependent accumulation of infected red blood cells in deep organs during experimental cerebral malaria. PLoS One 6: e18720. 21494565 10.1371/journal.pone.0018720 PMC3073989

[11] Nie CQ, Bernard NJ, Norman MU, Amante FH, Lundie RJ, et al (2009) IP-10-mediated T cell homing promotes cerebral inflammation over splenic immunity to malaria infection. PLoS Pathog 5: e1000369. 19343215 10.1371/journal.ppat.1000369 PMC2658824

[12] Belnoue E, Potter SM, Rosa DS, Mauduit M, Gruner AC, et al (2008) Control of pathogenic CD8+ T cell migration to the brain by IFN-gamma during experimental cerebral malaria. Parasite Immunol 30: 544–553. 18665903 10.1111/j.1365-3024.2008.01053.x

[13] Amani V, Vigario AM, Belnoue E, Marussig M, Fonseca L, et al (2000) Involvement of IFN-gamma receptor-medicated signaling in pathology and anti-malarial immunity induced by Plasmodium berghei infection. Eur J Immunol 30: 1646–1655. 10898501 10.1002/1521-4141(200006)30:6<1646::AID-IMMU1646>3.0.CO;2-0

[14] Rudin W, Favre N, Bordmann G, Ryffel B (1997) Interferon-gamma is essential for the development of cerebral malaria. Eur J Immunol 27: 810–815. 9130629 10.1002/eji.1830270403

[15] Rudin W, Eugster HP, Bordmann G, Bonato J, Muller M, et al (1997) Resistance to cerebral malaria in tumor necrosis factor-alpha/beta-deficient mice is associated with a reduction of intercellular adhesion molecule-1 up-regulation and T helper type 1 response. Am J Pathol 150: 257–266. 9006341 PMC1858518

[16] Grau GE, Heremans H, Piguet PF, Pointaire P, Lambert PH, et al (1989) Monoclonal antibody against interferon gamma can prevent experimental cerebral malaria and its associated overproduction of tumor necrosis factor. Proc Natl Acad Sci U S A 86: 5572–5574. 2501793 10.1073/pnas.86.14.5572 PMC297664

[17] de Miranda AS, Lacerda-Queiroz N, de Carvalho Vilela M, Rodrigues DH, Rachid MA, et al (2011) Anxiety-like behavior and proinflammatory cytokine levels in the brain of C57BL/6 mice infected with Plasmodium berghei (strain ANKA). Neurosci Lett 491: 202–206. 21256928 10.1016/j.neulet.2011.01.038

[18] Togbe D, de Sousa PL, Fauconnier M, Boissay V, Fick L, et al (2008) Both functional LTbeta receptor and TNF receptor 2 are required for the development of experimental cerebral malaria. PLoS One 3: e2608. 18612394 10.1371/journal.pone.0002608 PMC2442868

[19] Engwerda CR, Mynott TL, Sawhney S, De Souza JB, Bickle QD, et al (2002) Locally up-regulated lymphotoxin alpha, not systemic tumor necrosis factor alpha, is the principle mediator of murine cerebral malaria. J Exp Med 195: 1371–1377. 12021316 10.1084/jem.20020128 PMC2193758

[20] Grau GE, Lou JN (1995) Experimental cerebral malaria: possible new mechanisms in the TNF-induced microvascular pathology. Soz Praventivmed 40: 50–57. 7900436 10.1007/BF01615662

[21] de Kossodo S, Grau GE (1993) Profiles of cytokine production in relation with susceptibility to cerebral malaria. J Immunol 151: 4811–4820. 8409439

[22] Grau GE, Fajardo LF, Piguet PF, Allet B, Lambert PH, et al (1987) Tumor necrosis factor (cachectin) as an essential mediator in murine cerebral malaria. Science 237: 1210–1212. 3306918 10.1126/science.3306918

[23] Achtman AH, Pilat S, Law CW, Lynn DJ, Janot L, et al (2012) Effective adjunctive therapy by an innate defense regulatory peptide in a preclinical model of severe malaria. Sci Transl Med 4: 135ra164. 10.1126/scitranslmed.3003515 22623740

[24] Serghides L, Kim H, Lu Z, Kain DC, Miller C, et al (2011) Inhaled nitric oxide reduces endothelial activation and parasite accumulation in the brain, and enhances survival in experimental cerebral malaria. PLoS One 6: e27714. 22110737 10.1371/journal.pone.0027714 PMC3218025

[25] Serghides L (2012) The Case for the Use of PPARgamma Agonists as an Adjunctive Therapy for Cerebral Malaria. PPAR Res 2012: 513865. 21772838 10.1155/2012/513865 PMC3135089

[26] John CC, Kutamba E, Mugarura K, Opoka RO (2010) Adjunctive therapy for cerebral malaria and other severe forms of Plasmodium falciparum malaria. Expert Rev Anti Infect Ther 8: 997–1008. 20818944 10.1586/eri.10.90 PMC2987235

[27] Charunwatthana P, Abul Faiz M, Ruangveerayut R, Maude RJ, Rahman MR, et al (2009) N-acetylcysteine as adjunctive treatment in severe malaria: a randomized, double-blinded placebo-controlled clinical trial. Crit Care Med 37: 516–522. 19114891 10.1097/CCM.0b013e3181958dfd PMC2731834

[28] Bienvenu AL, Ferrandiz J, Kaiser K, Latour C, Picot S (2008) Artesunate-erythropoietin combination for murine cerebral malaria treatment. Acta Trop 106: 104–108. 18359468 10.1016/j.actatropica.2008.02.001

[29] Day N, Dondorp AM (2007) The management of patients with severe malaria. Am J Trop Med Hyg 77: 29–35. 18165472

[30] Penet MF, Abou-Hamdan M, Coltel N, Cornille E, Grau GE, et al (2008) Protection against cerebral malaria by the low-molecular-weight thiol pantethine. Proc Natl Acad Sci U S A 105: 1321–1326. 18195363 10.1073/pnas.0706867105 PMC2234136

[31] Planche T, Krishna S (2005) The relevance of malaria pathophysiology to strategies of clinical management. Curr Opin Infect Dis 18: 369–375. 16148522 10.1097/01.qco.0000180161.38530.81

[32] Craig AG, Grau GE, Janse C, Kazura JW, Milner D, et al (2012) The role of animal models for research on severe malaria. PLoS Pathog 8: e1002401. 22319438 10.1371/journal.ppat.1002401 PMC3271056

[33] Renia L, Potter SM, Mauduit M, Rosa DS, Kayibanda M, et al (2006) Pathogenic T cells in cerebral malaria. Int J Parasitol 36: 547–554. 16600241 10.1016/j.ijpara.2006.02.007

[34] Hunt NH, Grau GE, Engwerda C, Barnum SR, van der Heyde H, et al (2010) Murine cerebral malaria: the whole story. Trends Parasitol 26: 272–274. 20382078 10.1016/j.pt.2010.03.006

[35] Hunt NH, Grau GE (2003) Cytokines: accelerators and brakes in the pathogenesis of cerebral malaria. Trends Immunol 24: 491–499. 12967673 10.1016/s1471-4906(03)00229-1

[36] Serhan CN, Krishnamoorthy S, Recchiuti A, Chiang N (2011) Novel anti-inflammatory--pro-resolving mediators and their receptors. Curr Top Med Chem 11: 629–647. 21261595 10.2174/1568026611109060629 PMC3094721

[37] Serhan CN (2007) Resolution phase of inflammation: novel endogenous anti-inflammatory and proresolving lipid mediators and pathways. Annu Rev Immunol 25: 101–137. 17090225 10.1146/annurev.immunol.25.022106.141647

[38] Aliberti J, Hieny S, Reis e Sousa C, Serhan CN, Sher A (2002) Lipoxin-mediated inhibition of IL-12 production by DCs: a mechanism for regulation of microbial immunity. Nat Immunol 3: 76–82. 11743584 10.1038/ni745

[39] Aliberti J, Serhan C, Sher A (2002) Parasite-induced lipoxin A4 is an endogenous regulator of IL-12 production and immunopathology in Toxoplasma gondii infection. J Exp Med 196: 1253–1262. 12417634 10.1084/jem.20021183 PMC2194099

[40] Bafica A, Scanga CA, Serhan C, Machado F, White S, et al (2005) Host control of Mycobacterium tuberculosis is regulated by 5-lipoxygenase-dependent lipoxin production. J Clin Invest 115: 1601–1606. 15931391 10.1172/JCI23949 PMC1136995

[41] Herb F, Thye T, Niemann S, Browne EN, Chinbuah MA, et al (2008) ALOX5 variants associated with susceptibility to human pulmonary tuberculosis. Hum Mol Genet 17: 1052–1060. 18174194 10.1093/hmg/ddm378

[42] Birnbaum Y, Ye Y, Lin Y, Freeberg SY, Huang MH, et al (2007) Aspirin augments 15-epi-lipoxin A4 production by lipopolysaccharide, but blocks the pioglitazone and atorvastatin induction of 15-epi-lipoxin A4 in the rat heart. Prostaglandins Other Lipid Mediat 83: 89–98. 17259075 10.1016/j.prostaglandins.2006.10.003

[43] Birnbaum Y, Ye Y, Lin Y, Freeberg SY, Nishi SP, et al (2006) Augmentation of myocardial production of 15-epi-lipoxin-a4 by pioglitazone and atorvastatin in the rat. Circulation 114: 929–935. 16908763 10.1161/CIRCULATIONAHA.106.629907

[44] Serhan CN, Maddox JF, Petasis NA, Akritopoulou-Zanze I, Papayianni A, et al (1995) Design of lipoxin A4 stable analogs that block transmigration and adhesion of human neutrophils. Biochemistry 34: 14609–14615. 7578068 10.1021/bi00044a041

[45] Palmer CD, Guinan EC, Levy O (2011) Deficient expression of bactericidal/permeability-increasing protein in immunocompromised hosts: translational potential of replacement therapy. Biochem Soc Trans 39: 994–999. 21787336 10.1042/BST0390994

[46] Walker J, Dichter E, Lacorte G, Kerner D, Spur B, et al (2011) Lipoxin a4 increases survival by decreasing systemic inflammation and bacterial load in sepsis. Shock 36: 410–416. 21701419 10.1097/SHK.0b013e31822798c1

[47] Aliberti J (2005) Host persistence: exploitation of anti-inflammatory pathways by Toxoplasma gondii. Nat Rev Immunol 5: 162–170. 15662369 10.1038/nri1547

[48] McBerry C, Gonzalez RM, Shryock N, Dias A, Aliberti J (2012) SOCS2-induced proteasome-dependent TRAF6 degradation: a common anti-inflammatory pathway for control of innate immune responses. PLoS One 7: e38384. 22693634 10.1371/journal.pone.0038384 PMC3367914

[49] Schwab JM, Chiang N, Arita M, Serhan CN (2007) Resolvin E1 and protectin D1 activate inflammation-resolution programmes. Nature 447: 869–874. 17568749 10.1038/nature05877 PMC2757086

[50] Takano T, Clish CB, Gronert K, Petasis N, Serhan CN (1998) Neutrophil-mediated changes in vascular permeability are inhibited by topical application of aspirin-triggered 15-epi-lipoxin A4 and novel lipoxin B4 stable analogues. J Clin Invest 101: 819–826. 9466977 10.1172/JCI1578 PMC508630

[51] Maddox JF, Hachicha M, Takano T, Petasis NA, Fokin VV, et al (1997) Lipoxin A4 stable analogs are potent mimetics that stimulate human monocytes and THP-1 cells via a G-protein-linked lipoxin A4 receptor. J Biol Chem 272: 6972–6978. 9054386 10.1074/jbc.272.11.6972

[52] Higgins SJ, Kain KC, Liles WC (2011) Immunopathogenesis of falciparum malaria: implications for adjunctive therapy in the management of severe and cerebral malaria. Expert Rev Anti Infect Ther 9: 803–819. 21905788 10.1586/eri.11.96

[53] Van den Steen PE, Geurts N, Deroost K, Van Aelst I, Verhenne S, et al (2010) Immunopathology and dexamethasone therapy in a new model for malaria-associated acute respiratory distress syndrome. Am J Respir Crit Care Med 181: 957–968. 20093644 10.1164/rccm.200905-0786OC

[54] Prasad K, Garner P (2000) Steroids for treating cerebral malaria. Cochrane Database Syst Rev CD000972. 10.1002/14651858.CD000972 PMC6532619 10796562

[55] Rampengan TH (1991) Cerebral malaria in children. Comparative study between heparin, dexamethasone and placebo. Paediatr Indones 31: 59–66. 1852471

[56] Hoffman SL, Rustama D, Punjabi NH, Surampaet B, Sanjaya B, et al (1988) High-dose dexamethasone in quinine-treated patients with cerebral malaria: a double-blind, placebo-controlled trial. J Infect Dis 158: 325–331. 3042874 10.1093/infdis/158.2.325

[57] Wyler DJ (1988) Steroids are out in the treatment of cerebral malaria: what's next? J Infect Dis 158: 320–324. 3042873 10.1093/infdis/158.2.320

[58] Willcox ML (2001) Salicylates, nitric oxide, malaria, and Reye's syndrome. Lancet 357: 1881–1882. 10.1016/S0140-6736(00)04982-5 11417574

[59] Keri JE, Thomas K, Berman B, Falabella A (2000) Purpura fulminans in a patient with malaria. Eur J Dermatol 10: 617–619. 11125325

[60] Morris T, Stables M, Hobbs A, de Souza P, Colville-Nash P, et al (2009) Effects of low-dose aspirin on acute inflammatory responses in humans. J Immunol 183: 2089–2096. 19597002 10.4049/jimmunol.0900477

