# Supplementary figures and images for: Lipoxin A4 and 15-Epi-Lipoxin A4 Protect against Experimental Cerebral Malaria by Inhibiting IL-12/IFN-γ in the Brain
Source: PLoS One. 2013 Apr 16;8(4):e61882. doi: 10.1371/journal.pone.0061882 (PMC3628580; doi:10.1371/journal.pone.0061882)

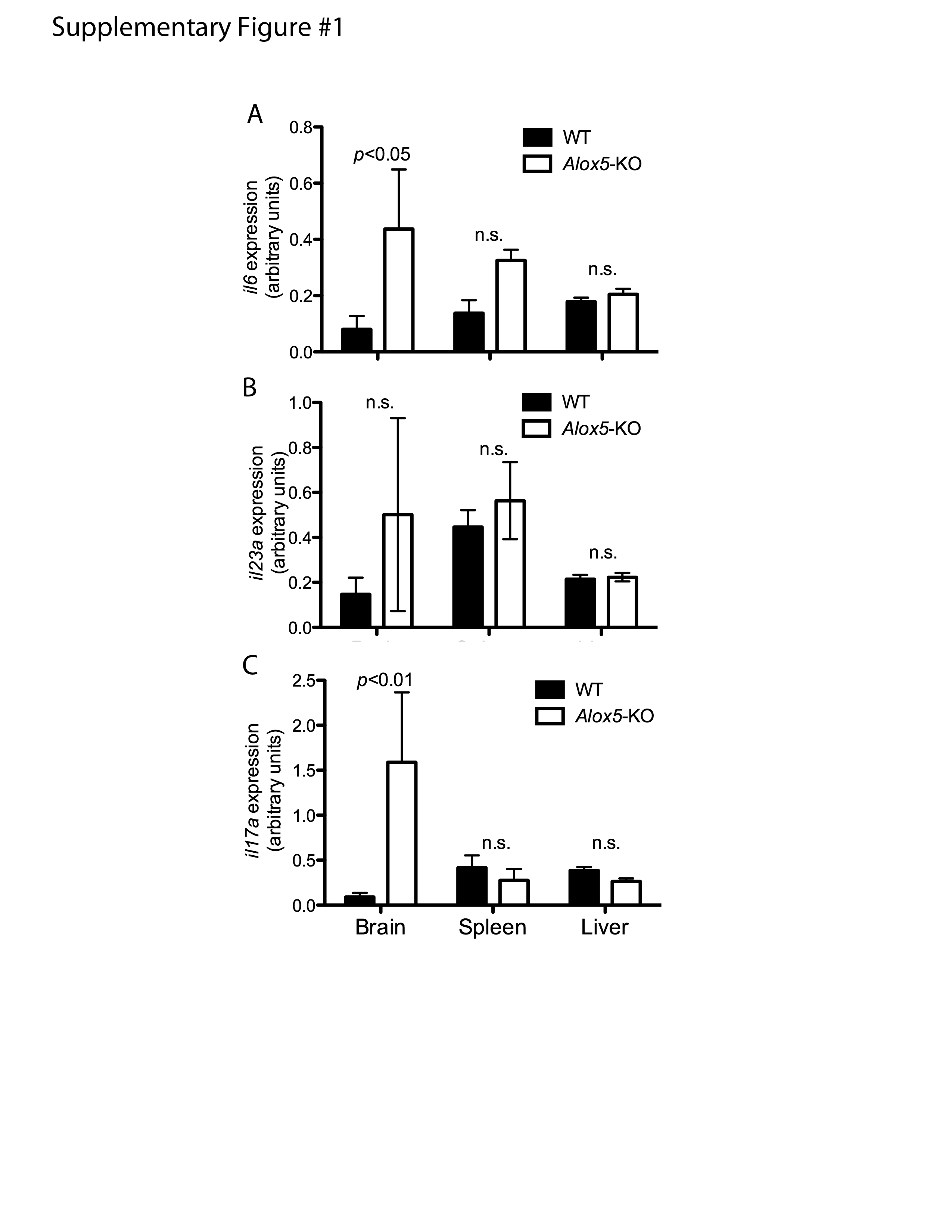

Supplement: Figure S1 — Enhanced il6 and il17A mRNA expression in P. berghei ANKA infected Alox5-deficient mice. C57Bl/6 WT and Alox5 −/− (n = 4 mice/group) mice were infected i.p. with P. berghei ANKA strain. Five days after infection, mice were sacrificed and brains, livers and spleens harvested, homogenized, total RNA extracted and reverse transcripted. Real-time RT-PCR was performed for determination of il6 (A), il23a (B) and il17a (C) expression. Data shown are representative of one out of three independent experiments performed. Statistical differences were determined using Mann Whitney test. (TIF) [file pone.0061882.s001.tif]
